# Pregnancy and kidney transplantation: optimized clinical and obstetric management to reduce maternal and perinatal complications

**DOI:** 10.1590/2175-8239-JBN-2025-0208en

**Published:** 2026-04-10

**Authors:** Annie Caroline Magalhães Santos, Rogério Caixeta Moraes de Freitas, Soubhi Kahhale, Rossana Pulcineli Vieira Francisco, Marco Aurélio Knippel Galletta

**Affiliations:** 1 Universidade Federal de Mato Grosso, Faculdade de Medicina, Departamento de Obstetrícia e Ginecologia, Cuiabá, MT, Brazil.; 2 Centro Universitário de Várzea Grande, Departamento de Obstetrícia e Ginecologia, Várzea Grande, MT, Brazil.; 3 Universidade de São Paulo, Faculdade de Medicina, Departamento de Obstetrícia e Ginecologia, Disciplina de Obstetrícia, São Paulo, SP, Brazil.; 4 Hospital e Maternidade São Luiz Star, Departamento de Obstetrícia de Alto Risco, São Paulo, SP, Brazil.

**Keywords:** Pregnancy, Renal Insufficiency, Chronic, Kidney Transplantation, Therapeutics, Pregnancy Outcome, Pregnancy Complications

## Abstract

**Objective:**

Review the literature to provide guidance on the appropriate clinical and obstetric management of pregnant women with kidney transplants to reduce maternal and perinatal complications.

**Methods:**

The PubMed and SciELO databases, which evaluated pregnancy and kidney transplantation, were searched, focusing on clinical and obstetric management. Other variables of interest were preconception counseling, fertility, the safety of immunosuppressants, maternal complications, perinatal complications, graft worsening during pregnancy, and postpartum guidance.

**Results:**

A total of 354 articles were identified, of which 76 were included in the analysis. Studies suggest that attempts to conceive should begin at least one year after kidney transplantation in the absence of graft rejection, with stable renal function (creatinine < 1.5 mg/dL and minimal proteinuria < 500 mg/24 h), with adjustment of the class and dose of immunosuppressants (preferably corticosteroids, azathioprine, cyclosporine, tacrolimus, and hydroxychloroquine). Preeclampsia prophylaxis with acetylsalicylic acid (81–50 mg/night) and calcium (1–1.5 g/day) should be universal in this group. Monitoring of renal function, fetal growth, and fetal vitality must be frequent.

**Conclusion:**

Pregnancy in kidney transplant recipients is challenging due to maternal and perinatal risks. However, the present review demonstrates that clinical and obstetric management by an experienced multidisciplinary team contributes to better maternal and perinatal outcomes in pregnancies among kidney transplant recipients.

## Introduction

Chronic kidney disease (CKD) estimates suggest that the early-stage disease affects 3 out of 100 pregnancies, whereas advanced stages affect 1 out of 750 pregnancies. Some of these patients experience loss of kidney function and require renal replacement therapy, such as hemodialysis and kidney transplantation^1,2^.

Patients with CKD may present blockade of the hypothalamic-pituitary-ovarian axis, with reduced serum estrogen levels and prolactin clearance, leading to anovulation, amenorrhea, and infertility. However, with advances in dialysis techniques and an increase in the rate of kidney transplants, conception rates have become more frequent^3^. The fertility rates in women undergoing kidney transplants and those on hemodialysis are approximately 1/10 and 1/100, respectively^4^.

Care for pregnant women receiving kidney transplantation represents a challenge, not only because of the physiological changes in the renal system during pregnancy, but also because of the risk of graft rejection, in addition to the greater risk of concomitant comorbidities, such as cardiovascular disorders and diabetes^5,6^.

Meta-analyses reiterate the impact of kidney disease during pregnancy, with a range of complications; therefore, it is important to understand the best time for conception, as well as the most appropriate clinical and obstetric management, to optimize maternal and fetal prognosis after kidney transplantation^5,7^.

Although it is considered a high-risk obstetric situation, pregnancy in kidney transplant patients is not contraindicated. However, it requires adequate guidance so that conception occurs at a time of clinical stability of the underlying disease and other comorbidities, with the use of immunosuppressants in safe doses aimed at reducing the risk of compromising renal function and consequent graft loss. Furthermore, multidisciplinary monitoring with obstetricians, nephrologists, nutritionists, and neonatologists is essential for the surveillance and adequate management of pregnant women who have undergone kidney transplants^8^.

Despite the increased fertility rate in kidney transplant recipients, there is still limited evidence, especially regarding appropriate clinical and obstetric management for better obstetric and neonatal outcomes^8,9,10^. Therefore, the aim of this review is to explain the current recommendations related to pregnancy in women with kidney transplantation, highlighting new consensuses and clarifying uncertainties about clinical and obstetric management, as well as treatment details, to reduce possible complications.

## Method

To prepare this integrative review, studies that addressed pregnancy and kidney transplantation were included, with a focus on clinical and obstetric management. We included studies available in PubMed and the SciELO databases, published up to December 2024. The following descriptors were used: pregnancy, kidney transplantation, and chronic kidney disease. The abstracts of 354 articles were evaluated, of which 76 were selected, including meta-analyses and systematic reviews. We sought to select articles that focused on the following aspects: preconception counseling in post-transplant pregnancy; clinical and obstetric management and maternal and perinatal complications; worsening of graft function during pregnancy; and postpartum care and breastfeeding.

### Fertility and Conception Following Kidney Transplantation

In addition to the recovery of kidney function, one of the greatest benefits of kidney transplantation for women with CKD is the recovery of reproductive and sexual function and fertility, which occurs approximately six months after transplantation^11,12^. Pregnancy in kidney transplant recipients is relatively rare, approximately five cases per 100,000 births^11^. Kidney transplantation may increase the chance of live birth in pregnant women who receive a transplant tenfold compared to those on dialysis^12,13^.

The American Society of Transplantation, the Italian Society of Nephrology, the European Best Practice Guidelines, and KDIGO (Kidney Disease: Improving Global Outcomes) suggest that women should be advised to wait at least one year after the transplant^9,10,14^, and, preferably, up to two years, before conception to avoid graft dysfunction, rejection or failure, infections, and the teratogenic effects of immunosuppressants^10,14,15^.

The American Society of Kidney Transplantation^14,15^ establishes the following recommendations for a pregnancy with a lower risk of complications:

- Absence of rejection episodes for at least one year;
- Stable renal function (creatinine < 1.5 mg/dL) and absent or minimal proteinuria (< 500 mg/24 h);
- Absence of infections that could be harmful to the fetus (cytomegalovirus and toxoplasmosis);
- Use of non-teratogenic immunosuppressants;
- Consideration of additional factors: maternal age; therapeutic adherence; comorbidities that may affect pregnancy and transplant function^14,15^.

Experts recommend that women with CKD wait for a kidney transplant before trying to get pregnant. However, it should be emphasized that, even after transplantation, pregnancy remains at high risk of complications (preeclampsia, opportunistic infections, prematurity, fetal growth restriction [FGR], and perinatal mortality), requiring close follow-up during prenatal care and supervision by an experienced multidisciplinary team^16^.

### Safety of Immunosuppressants and Other Medications

Immunosuppressants are used in pregnant women with autoimmune diseases (lupus, primary glomerulonephritis, and others) to control the disease and maintain remission, as well as in kidney transplant recipients to avoid graft rejection^17^. Immunosuppressants cross the placental barrier, and, therefore, it is essential to assess the risk of teratogenicity and fetal toxicity^10^.

Mycophenolate mofetil and cyclophosphamide are teratogenic and must be replaced, ideally three months before pregnancy^18,19^. They can increase the risk of malformations such as congenital facial, cardiac, and visceral anomalies and diaphragmatic hernia, in addition to increasing the risk of spontaneous abortion^17,20,21,22^.

Corticosteroids, azathioprine, and calcineurin inhibitors (cyclosporine and tacrolimus) have a good safety profile in pregnancy^18,21^. Azathioprine can cause severe bone marrow suppression in some pregnant women who have received a kidney transplant, requiring close monitoring^20,23^. The pharmacokinetics of calcineurin inhibitors change during pregnancy, requiring a dose increase of 20 to 25% to achieve adequate concentrations^24,25^. Sirolimus and everolimus are contraindicated during pregnancy due to their antiproliferative effect, which may affect fetal development^24^.

Of the corticosteroids, prednisone is the safest, as only a small fraction crosses the placental barrier. On the other hand, women who take doses greater than 1–2 mg/kg/day throughout pregnancy and close to delivery may present suppression of the hypothalamic-pituitary-adrenal axis, and the administration of intravenous corticosteroids at the time of delivery is recommended to avoid complications. Chronic use of corticosteroids increases the risk of adrenal insufficiency, hypertension, and gestational diabetes. Additionally, there is still a risk of thymic hypoplasia in childhood. For all these reasons, such use must be undertaken with caution^17,25^.

Immunobiological agents such as rituximab, eculizumab, and belimumab differ in structure and half-life; although placental transfer is limited during organogenesis, it increases exponentially throughout pregnancy^26^. Information on the safety of these agents in pregnancy is scarce, and there are few specific recommendations and studies during the preconception period, pregnancy, and lactation.

Hydroxychloroquine is an immunomodulator used in lupus to prevent disease flares, with known safety data during pregnancy and lactation; it crosses the placental barrier but is not associated with fetal toxicity. There is also evidence of improved placentation and prevention of FGR, as well as atrioventricular block and congenital lupus^19,27^.

Medication choices should be individualized, considering the risks of opportunistic infections, structural malformations, spontaneous abortion, and preterm birth versus the risk of maternal disease recurrence due to interruption of these therapeutic agents^20,26^. Table 1 presents the immunosuppressants and medications used in patients with CKD and kidney transplants, and their safety during pregnancy and lactation.

**Table 1:** Immunosuppressants and other drugs used in patients with ckd and kidney transplantation during the pregnancy–puerperal period

Drugs	Conception Safe/Unsafe	Safe/Unsafe	Pregnancy and maternal effects	Fetal effects	Lactation safe/Unsafe
IMMUNOSUPRESSIVE DRUGS					
Corticosteroids	Safe	Safe (C)	Higher risk of bone loss, diabetes, infection, preterm rupture of membranes	No teratogenicity (no association with orofacial clefts in recent studies)	Safe (monitor newborn)
Azathioprine	Safe	Safe (D)	Bone marrow suppression, hepatotoxicity, hyperkalemia, worsening hypertension and nephrotoxicity	No metabolization by the fetal liver	Safe
Cyclosporine	Safe	Safe (C)	Risk of hypertensive, hyperglycemic, and nephrotoxic effects	No teratogenicity (reports of FGR and SGA)	Safe
Tacrolimus	Safe	Safe (C)	Similar to cyclosporine	No teratogenicity	Safe
Hydroxychloroquine	Safe	Safe (C)	Withdrawal may trigger lupus flare	No teratogenicity	Safe
TO BE AVOIDED					
mTOR inhibitors (Sirolimus, Everolimus)	Unsafe	Unsafe (C)	Hyperlipidemia, hyperglycemia, nephrotoxicity	Unclear (toxic in animal studies)	Unclear
Mycophenolate	Unsafe (♀ and ♂ stop > 6 weeks before conception)	Unsafe (D)	Gastrointestinal symptoms and dose-related bone marrow suppression	Teratogenic. Severe fetal cardiovascular and cranial malformations	Unsafe (excreted in breast milk)
Methotrexate	Unsafe (stop > 3 months before conception)	Unsafe (X)	Hepatotoxicity, gastrointestinal symptoms, alopecia, bone marrow suppression	Teratogenic	Unsafe (excreted in breast milk)
Cyclophosphamide	Unsafe (♀ and ♂ stop > 3 months before conception)	Unsafe (D)	Affects ovarian function and fertility	Teratogenic. Congenital abnormalities of the skull, ear, face, limbs, and visceral organs, cytopenia	Unsafe (excreted in breast milk. Discontinue breastfeeding during treatment and for 36 hours after its completion)
BIOLOGIC AGENTS					
Rituximab	Unclear (manufacturer advises discontinuation 1 year before conception)	Unclear	Active passage in the 2nd and 3rd trimesters. Administration before or in early pregnancy	Avoid unless potential benefits outweigh risks (potential risk of neonatal B-cell depletion)	Unclear
Eculizumab	Unclear	Unclear	Monitor for increased dosage requirements	Active passage in the 2nd and 3rd trimesters. No teratogenicity reported. Studies to date showed no teratogenicity	Unclear
Belimumab	Unclear (stop > 4 months before conception)	Unclear	Active passage in the 2nd and 3rd trimesters. Administration before or in early pregnancy	Limited data. Avoid unless potential benefits outweigh risks	Unclear
OTHER DRUGS						
Aspirin	Safe	Safe (C)	Decreases the risk of preeclampsia and FGR	No teratogenicity	Safe	
Low-molecular-weight heparin	Safe	Safe (C)	Decrease the risk of VTE	No teratogenicity	Safe	
Allopurinol	Safe	Safe (C)	None	No teratogenicity	Excreted in breast milk. No reported adverse effects
Iron	Safe	Safe (B)	None	No teratogenicity	Safe	
Erythropoietin	Safe	Safe (C)	Risk of hypertension	No teratogenicity	Safe	
BIOLOGIC AGENTS					

Note – Modified from Gouveia et al.^51^.

Armenti et al.^28^ reported a rate of 3 to 5% of congenital malformations in kidney transplant recipients. However, no association with congenital malformations was observed with the use of cyclosporine and tacrolimus^11,28^.

### Obstetric Management and Prenatal Care in Kidney Transplant Patients

In prenatal care, follow-up should involve more frequent consultations: every 3–4 weeks up to 28 weeks, every two weeks from 28 to 34 weeks, and then weekly. Assessments of fetal growth and development should be rigorous, with serial ultrasonography. From 20 weeks onwards, Doppler velocimetry should be performed, and after 34 weeks, biophysical profile and cardiotocography should be included to monitor fetal well-being^29^.

Rigorous screening for anemia should be carried out, with monthly hemoglobin and hematocrit measurements, instituting treatment with iron salts and erythropoietin when necessary. Furthermore, folic acid supplementation at a dose of 5 mg/day is recommended to prevent neural tube defects in the first trimester. Subsequently, folic acid should be maintained throughout pregnancy and combined with cyanocobalamin, 1 to 2 mg/day, to prevent megaloblastic anemia^8^.

It is recommended to use acetylsalicylic acid (ASA) at low doses (81–150 mg) in pregnant women with kidney transplantation. Treatment should be initiated at 12 weeks and discontinued at 36 weeks of gestation to reduce the risk of early preeclampsia and improve placentation in patients with positive factors for autoimmunity^19,30,31^. In countries with low intake of calcium-rich foods, calcium supplementation of 1–1.5 g/day is indicated to reduce the risk of preeclampsia^19^.

In pregnant women with lupus and reactive antiphospholipid antibodies, in addition to ASA, it is necessary to associate low molecular weight heparin at a prophylactic dose to prevent adverse obstetric and fetal outcomes^32,33^.

It is estimated that there is a 14.6% to 42% increase in the risk of urinary tract infection in these women, as well as an increased risk of progression to severe forms of pyelonephritis. Therefore, a monthly urine culture should be performed. Accordingly, prophylactic antibiotics are recommended after the first episode of urinary tract infection to prevent recurrence^7^. Evaluation of renal function should be early and serial (monthly), with serum measurements of urea and creatinine, as well as 24-hour proteinuria or the protein-to-creatinine ratio^34^. In addition, CMV DNA PCR should be performed monthly, as studies show that around 40% of CMV infections in immunosuppressed pregnant women result in fetal transmission, with 10% to 15% presenting severe involvement^35^. If seroconversion or reactivation of CMV infection occurs during pregnancy, treatment with ganciclovir or valacyclovir is recommended^36^.

Live attenuated vaccines are contraindicated in solid organ transplant patients. However, vaccination of pregnant women with kidney transplants is similar to that of other pregnant women, with vaccines against influenza, hepatitis B, diphtheria, tetanus, and acellular pertussis (DTaP) being recommended^37^.

Vaginal birth is not contraindicated due to the location of the kidney in the pelvic cavity. However, studies reveal high rates of cesarean section in kidney transplant patients, mainly due to complications such as preeclampsia and FGR^5,13^. The route of delivery is indicated by obstetric criteria, and it is suggested that labor induction be carried out at 38 weeks provided that there are no obstetric complications^5,38^.

When a cesarean section is indicated, an abdominal ultrasound should be performed to assess the correct location of the graft, with adequate surgical planning, avoiding trauma to the graft and inadvertent ligation of the ureter, which can occur in up to 2% of cases^39^.

Thus, despite the risks involved, most studies indicate that it is possible to have successful pregnancies after kidney transplantation. The main behaviors that result in better maternal and perinatal outcomes include:

- Close monitoring of the function of the grafted kidney;
- Screening and adequate treatment of infections, mainly of the genitourinary tract;
- Early initiation of medications that can prevent preeclampsia (ASA and calcium salts);
- Strict control of blood pressure levels;
- Safe immunosuppressants at adjusted doses;
- Supervision of fetal growth and surveillance of fetal well-being^8^.

**Figure 1:**
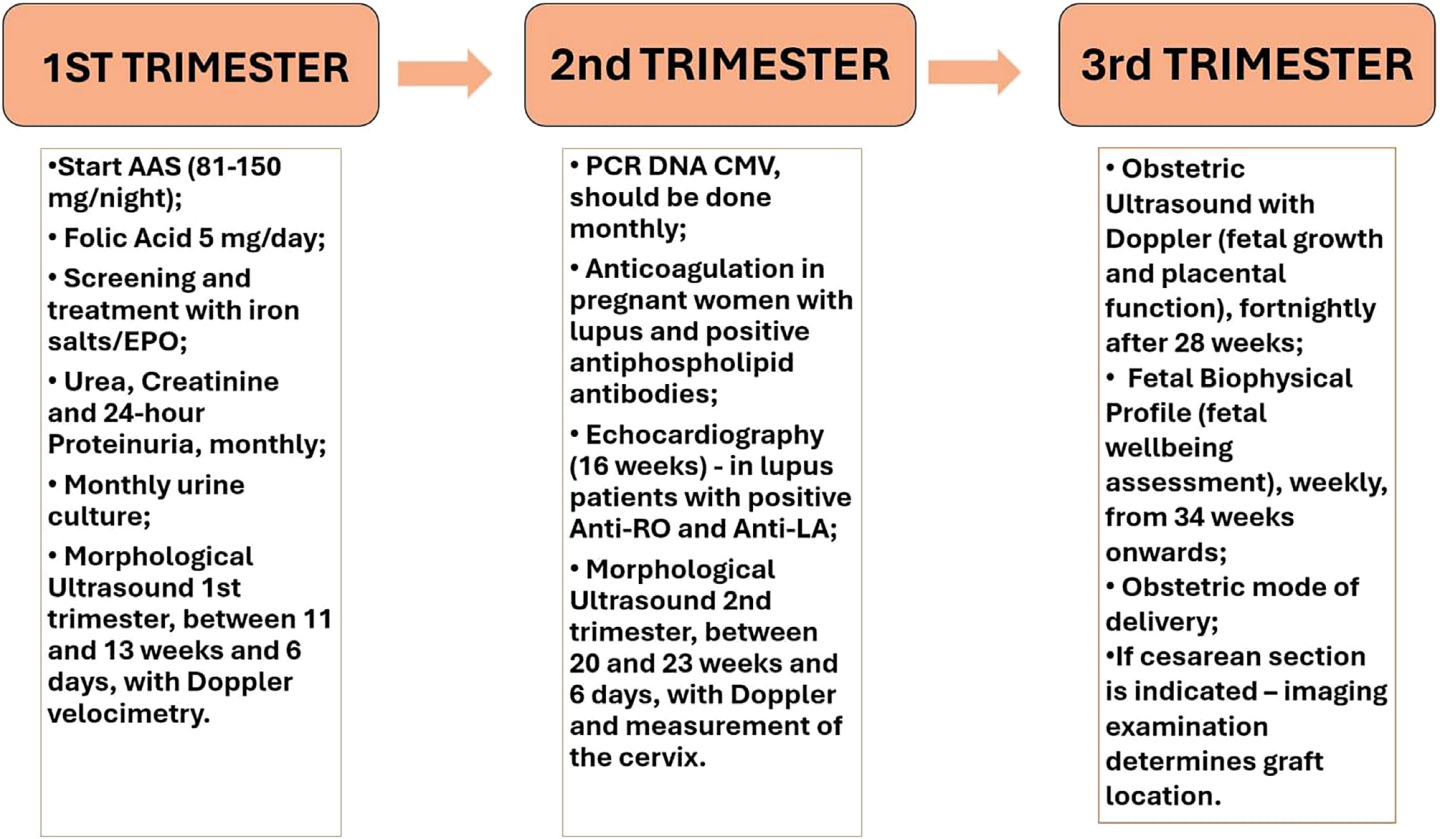
Clinical and obstetric management in pregnant women with kidney transplants.

Figure 1 presents a schematic structure of the clinical and obstetric management in pregnant women with kidney transplants.

Note – Prepared by the authors.

### Maternal Complications in Kidney Transplantation

One of the main predictors of maternal and fetal prognosis and outcomes is the lack of stable pre-pregnancy renal function, with elevated serum creatinine (>1.5 mg/dL)^13,40,41^.

In kidney transplant recipients, the live birth rate is close to 75–80%, higher than in dialysis. However, despite the restoration of renal function, these pregnancies still have a higher incidence of complications^12,42,43^.

The most common complications are hypertension (24.2–54.2%), preeclampsia (21.5–31%), prematurity (43.1–65.4%), small-for-gestational-age newborns (54%), and acute graft rejection during pregnancy (1–9.4%) and int the postpartum period (1.3%)^10,12,42,43,44^.

Furthermore, higher rates of cesarean sections are reported (53–72%)^43,44^. These rates vary greatly in international cohorts on transplants and pregnancy, such as the American cohort by Shah et al.^44^ (35.4%) and the Chinese cohort by Ma et al.^45^ (94.1%).

In pregnant women with kidney transplants, the diagnosis of preeclampsia is complex and challenging, as the majority of these patients are already hypertensive, and it is common for proteinuria to develop during pregnancy due to the underlying disease, even in the absence of preeclampsia^46,47,48^. Stepan et al. (2020) demonstrated that angiotensin II promotes the production of soluble fms-like tyrosine kinase-1 (sFlt-1) in proximal tubular cells. Evidence suggests that the use of these biomarkers, associated with uteroplacental Doppler flow, may be an auxiliary tool for the differential diagnosis of preeclampsia in pregnant women with CKD. However, owing to costs and limited availability in clinical practice, further studies are necessary^48^.

Chewcharat et al.^16^, analyzing data from their cohort of 295 pregnant women with kidney transplantation and 405 with CKD stages 3–5, indicated a slightly lower prevalence of preeclampsia (34.6%) in pregnant women with kidney transplants compared to those in final stages of CKD (48.4%), but higher than that of pregnant women without kidney disease (4.4%). They also concluded that there was a threefold higher risk of prematurity and a twofold higher risk of FGR in kidney transplant recipients^16^. Similarly, a meta-analysis by Shah et al.^44^ demonstrated a preeclampsia rate six times higher in kidney transplant recipients than in the general U.S. population (21.5% vs. 3.8%)^16,44^. Regarding data on pregnancies in Brazilian kidney transplant recipients, the study by Radaelli et al.^49^ demonstrated a higher rate of preeclampsia (42%).

For women who underwent kidney transplantation in childhood, an association with congenital kidney malformations is possible and, therefore, urogenital tract abnormalities should be excluded. In that regard, changes in the uterine and cervical cavities must be tracked, as they can increase the risk of spontaneous premature birth^50^, since kidney transplant recipients already have a 30.6% higher risk of delivery occurring at < 37 weeks than the general population^11^.

Due to the use of immunosuppressants and corticosteroids, kidney transplant recipients are at greater risk of urinary tract infections (UTIs), chorioamnionitis, and endometritis than other pregnant women^11,51^. Studies have shown a higher risk (14.6–42%) of UTIs^51,52^. A recent meta-analysis by Souza et al.^52^ revealed that the prevalence of UTI among Latin American pregnant women was 7.54%; this rate was slightly higher when considering asymptomatic bacteriuria, estimated at 18.45%^52^. Urinary tract infection and acute pyelonephritis are the main infectious complications in these women. Therefore, treatment must be assertive, as it reduces low birth weight and the risk of prematurity. It is mandatory to evaluate renal function during antibiotic therapy and after one week of treatment^53^.

Akcay et al.^42^ compared transplanted women who became pregnant more than two years after transplantation with transplanted women who did not become pregnant. Of the 29 pregnancies, 72.4% resulted in live births, and 38% of patients were diagnosed with preeclampsia. In contrast, 33% had proteinuria > 300 mg/dL but did not develop preeclampsia. Almost all births (90%) were cesarean sections, with 38% premature births and 19% of newborns small for gestational age. There was a fivefold increase in the probability (95% CI: 1,247–20,781; p < 0.02) of worsening renal function one year after pregnancy, compared with the control group, with at least a 25% drop in the glomerular filtration rate, suggesting that pregnancy compromises long-term renal function of the graft^42^.

**Figure 2:**
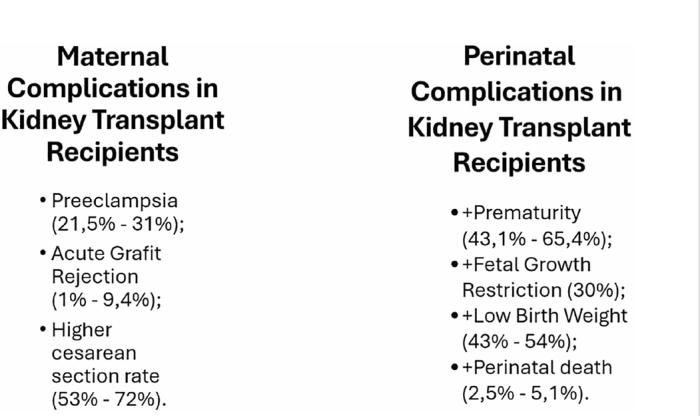
Maternal and perinatal complications in kidney transplant patients.

Meta-analyses suggest that pregnancy occurring within the first year after transplantation is associated with worse maternal outcomes^54,55^. Conversely, Australian and New Zealand studies have not demonstrated an increase in mortality during the pregnancy–puerperal cycle^46^. Figure 2 presents the main maternal complications observed in pregnant women with kidney transplants.

Note – Prepared by the authors.

### Perinatal Complications in Kidney Transplant Recipients

Compared to the general population, pregnancies in women with kidney transplants present higher rates of preterm, small for gestational age, and low birth weight newborns, leading to more frequent admissions to the neonatal intensive care unit (NICU). Thus, such outcomes result in higher rates of perinatal morbidity and mortality in these women^7,41,56,57,58^. Furthermore, intrauterine exposure to immunosuppressants contributes to an increase in infections in the first year of life^5^.

The United States National Transplant Registry demonstrated live birth rates in kidney transplant recipients ranging from 71% to 76%^56^. International cohorts, such as those of Shah et al.^44^ (72.9%), Mustafa et al.^59^ (78.5%), and the Brazilian cohort of Radaelli et al.^49^ (56.7%), showed similar results.

In studies carried out in the United States with kidney transplant recipients, births before 37 weeks range from 43.1% to 65.4%^7,44^. Shah et al.^44^ demonstrated that prematurity rates in this group are highest in South America (55%) and lowest in North America (35.4%)^7,44^.

The birth weight of children of kidney transplant mothers is, on average, lower than that of the general U.S. population: 2,470 g vs. 3,389 g^7,44^. On the other hand, Arab et al.^11^ demonstrated a high rate of FGR (30%). Maternal anemia is common in kidney transplant patients and may be one of the explanations for this association with FGR. Furthermore, even with kidney transplantation, the progression of vasculopathy in patients with diabetes and systemic vasculitis is not reduced, which may contribute to FGR and low birth weight^11^. Studies show that the rates of low-birth-weight newborns (weight < 2,500 g) are between 43% and 54% in these patients^42^. Shah et al.^44^ also concluded that there is a threefold higher risk of prematurity and a twofold higher risk of fetal growth restriction in transplant recipients^44^.

The stage of CKD and hypertension are important determinants of results^41^. Kidney transplant patients with an estimated glomerular filtration rate greater than 90 mL/min/1.73 m^2^ have worse outcomes compared to patients with stage 1 CKD; these differences level off when only CKD patients affected by glomerulonephritis or systemic diseases (“progressive CKD”) are compared with kidney transplant recipients (KTRs). In the multivariate analysis, the risk of preterm and early preterm delivery was linked with CKD stage (2–5 vs. 1: relative risk [RR] 3.42 and 3.78, respectively) and hypertension (RR 3.68 and 3.16, respectively), while no difference was associated with whether the patient was a KTR or had CKD^41^.

It is estimated that the risk of miscarriage is lower than that of the general population (14–15.4% vs. 17.1%)^11^. Perinatal deaths range from 2.5% to 5.1%, a rate higher than that of the general population in the United States (0.6%)^7,44^. The perinatal mortality rate is fourfold higher in kidney transplant recipients than in the general population, which may be attributed to FGR and prematurity^18^. This rate is higher in patients who become pregnant within the first two years after transplantation compared with those who become pregnant afterwards^60^. Figure 2 presents the main perinatal complications observed in pregnant women with kidney transplants.

Bachmann et al.^61^ followed children of women with kidney transplants from birth to two years of age and demonstrated that anthropometric measurements showed similar trajectories, regardless of whether births were premature or full term, and without changes in neuropsychomotor development^61^. Nulman et al.^62^ also did not find delays in neuropsychomotor development and behavior in children followed up to eight years of age who were exposed to intrauterine cyclosporine, compared to healthy female controls^62^.

### Worsening Graft Function During Pregnancy

Pregnancy in women with stable renal function does not increase the risk of graft rejection^63^. On the other hand, kidney transplant recipients without stable renal function have a risk of acute rejection of 1.3–9.4%^47,63^ and graft loss of 5.6–9.2%^44,63^ after pregnancy, especially if the pregnancy occurs less than one year after transplantation^10^. Recent literature demonstrates that higher rates of loss of graft function occur in women with creatinine > 1.5 mg/dL. Therefore, counseling kidney transplant recipients regarding the longevity of the graft is essential when considering pregnancy^13,40^.

The use of immunosuppressants at an adequate dose is directly linked to maintaining stable graft function, with a lower chance of rejection. Cyclosporine and tacrolimus are two of the most used agents and should be monitored serially during pregnancy, as their serum levels may decrease due to hemodilution and increased hepatic metabolism, requiring readjustments to reach therapeutic concentrations. Serum dosing should be performed monthly in the first and second trimesters and weekly in the third trimester of pregnancy^64^.

Akcay et al.^42^ demonstrated that long-term worsening of renal graft function is related to the persistence of high levels of proteinuria in the first three months postpartum, as well as high serum creatinine levels in the first six months postpartum. Furthermore, the time between transplantation and conception appears to influence the risk of deterioration in renal function^42^.

Records from the United States National Transplant Center indicate that women with loss of kidney function during or after pregnancy have preconception creatinine values higher than those with stable kidney function (1.5 ± 0.6 vs. 1.3 ± 0.4 mg/dL). On the other hand, African American women have a higher risk of losing kidney function if they become pregnant less than two years after transplantation (13%), compared to White women (5%)^65^.

Preconception hypertension appears to be one of the main predictors of renal function decline and long-term risk of rejection. It is estimated that 25% to 40% of women with solid organ transplants are already hypertensive before pregnancy^39^. Some factors related to this fact would be the vascular toxicity of calcineurins, corticosteroid-induced hypertension, and the vasopressor effect resulting from renal ischemia^64,66^.

Creatinine levels are significantly higher in hypertensive women prior to pregnancy^67,68^. Moderate to severe graft dysfunction is defined by creatinine > 1.4 mg/dL and 1.9 mg/dL, respectively^14,65,69^. Proteinuria > 1 g/24 h, before or during pregnancy, is also related to greater impairment of graft function^70^.

During pregnancy, the main cause of elevated serum creatinine is the presence of preeclampsia. However, it may also be related to urinary tract infection and obstruction or tacrolimus toxicity. However, any increase in creatinine must be immediately investigated and appropriately addressed to avoid progressive worsening of renal function, and the risk of graft loss^15^.

Women with kidney transplants and elevated levels of donor-specific anti-HLA (human leukocyte antigen) antibodies constitute a group at higher risk for renal dysfunction and graft loss. It is not known whether pregnancy acts as a trigger for this process or whether it is related to the time elapsed since transplantation^60^.

Kidney assessment can be improved by evaluating renal blood flow with Doppler velocimetry of the graft vessels, measuring flow velocity and the resistance index (RI). In kidneys with preserved function, RI decreases throughout pregnancy, mainly between 13 and 16 weeks, with a tendency to increase at the end of pregnancy. RI values > 0.8 present an unfavorable prognosis and may indicate the establishment of graft nephropathy, renal obstruction, pyelonephritis, or preeclampsia, requiring further investigation and an assertive approach to avoid graft damage^8^.

The rate of transplant rejection during pregnancy is low, around 4.2% in the American population, according to a recent meta-analysis^5^. During pregnancy, there is a mechanism of physiological immune tolerance, with the involvement of HLA-G molecules, which inhibit T lymphocytes, natural killer (NK) cells, and antigen-presenting cells^24^. If rejection is suspected, renal biopsy should be considered after fetal viability (> 25 weeks), and if gestational age is advanced (> 34 weeks), empirical treatment with methylprednisolone and consideration of pregnancy termination are suggested^46^.

### Postpartum Care and Breastfeeding

Studies show a twofold risk of postpartum hemorrhage (PPH) and the need for blood transfusions in postpartum women with kidney transplants. There are theories that suggest that immunosuppressants have a detrimental effect on the remodeling of uterine arterioles, reducing contractility and myotamponade, thereby increasing the risk of PPH^71,72^. Furthermore, the use of antibiotics after cesarean section is suggested due to the greater risk of infection associated with the use of immunosuppressants^11^.

There is evidence of excretion of immunosuppressive drugs in breast milk; however, the benefits of breastfeeding appear to outweigh the risks of exposure^73^. Tacrolimus, cyclosporine, azathioprine, and prednisone are considered safe during pregnancy and breastfeeding^71,72,73^.

### Pre-Conception Counseling

Contraception should be recommended for all women with CKD of reproductive age who are sexually active and do not wish to become pregnant. Combined estrogen-containing contraceptives are contraindicated due to increased cardiovascular and thromboembolic risk. Medroxyprogesterone acetate should be avoided due to possible reduction in bone mass. Progestogen-only pills, etonogestrel subdermal implants, and intrauterine devices are recommended^1,74^.

There is no evidence to support that intrauterine devices are less effective in kidney transplant recipients due to immunosuppression, nor that they are associated with an increased risk of pelvic inflammatory disease^75^.

A preconception consultation with a multidisciplinary team is essential to adjust immunosuppressants and vitamin supplementation and provide adequate guidance on prognosis. In addition, it is necessary to start prenatal care in a tertiary service with a team experienced in the management of high-risk pregnancies.

The replacement of teratogenic immunosuppressants (mycophenolate mofetil and cyclophosphamide) with safer classes (azathioprine, cyclosporine, and tacrolimus) and the suspension of embryotoxic medications during pregnancy should be carried out at least 90 days before conception^76^.

Therefore, guidance on the risks of worsening graft function, reduced graft longevity, and permanent loss of graft function are essential pillars of preconception counseling^57^.

## Conclusion

Post-kidney transplant pregnancies have increased in recent decades, and conception attempts should be planned at least one year after transplantation, in the absence of signs of graft rejection, to allow adjustment of immunosuppression and reduce the risk of graft loss. The couple should be informed that, although maternal and perinatal complications are lower in kidney transplant recipients than in pregnant women on hemodialysis, these pregnancies have a higher risk of preeclampsia, prematurity, low birth weight, cesarean section, and perinatal death. However, even though it is considered a high-risk pregnancy, if conception is planned at a time of maternal clinical stability, with safe immunosuppressants and obstetric management carried out by an experienced multidisciplinary team, the chance of a successful pregnancy becomes an achievable goal.

## Data Availability

All information in this review was collected from the references cited herein.
