# Supplementary material for: Pregnancy and kidney transplantation: optimized clinical and obstetric management to reduce maternal and perinatal complications
Source: J Bras Nefrol. 2026 Apr 10;48(3):e20250208. doi: 10.1590/2175-8239-JBN-2025-0208en (PMC13076013; doi:10.1590/2175-8239-JBN-2025-0208en)
Supplement: Translation

# Translation: Gravidez e transplante renal: manejo clínico e obstétrico otimizado para reduzir complicações maternas e perinatais

**DOI:** 10.1590/2175-8239-JBN-2025-0208pt

**Authors:** Annie Caroline Magalhães Santos, Rogério Caixeta Moraes de Freitas, Soubhi Kahhale, Rossana Pulcineli Vieira Francisco, Marco Aurélio Knippel Galletta

**Affiliations:** 1 Universidade Federal de Mato Grosso, Faculdade de Medicina, Departamento de Obstetrícia e Ginecologia, Cuiabá, MT, Brasil.; 2 Centro Universitário de Várzea Grande, Departamento de Obstetrícia e Ginecologia, Várzea Grande, MT, Brasil.; 3 Universidade de São Paulo, Faculdade de Medicina, Departamento de Obstetrícia e Ginecologia, Disciplina de Obstetrícia, São Paulo, SP, Brasil.; 4 Hospital e Maternidade São Luiz Star, Departamento de Obstetrícia de Alto Risco, São Paulo, SP, Brasil.

**Keywords:** Gravidez, Insuficiência Renal Crônica, Transplante Renal, Terapêutica; Desfecho da Gravidez, Complicações da Gravidez

## Abstract

**Objetivo:**

Revisar a literatura para fornecer orientações sobre o manejo clínico e obstétrico adequado de gestantes submetidas a transplante renal, visando reduzir complicações maternas e perinatais.

**Métodos:**

As bases de dados PubMed e SciELO foram consultadas, nas quais se identificaram estudos que avaliaram gravidez e transplante renal, com foco no manejo clínico e obstétrico. Outras variáveis de interesse foram o aconselhamento pré-concepcional, a fertilidade, a segurança dos imunossupressores, as complicações maternas, as complicações perinatais, a piora do enxerto durante a gravidez e o acompanhamento pós-parto.

**Resultados:**

Foram identificados 354 artigos, dos quais 76 foram incluídos na análise. Os estudos sugerem que as tentativas de concepção devem começar pelo menos um ano após o transplante renal, na ausência de rejeição do enxerto, com função renal estável (creatinina < 1,5 mg/dL e proteinúria mínima [< 500 mg/24 h]), com ajuste de classe e dose do imunossupressor (preferencialmente corticosteroides, azatioprina, ciclosporina, tacrolimo e hidroxicloroquina). A profilaxia da pré-eclâmpsia com ácido acetilsalicílico (81–50 mg/noite) e cálcio (1–1,5 g/dia) deve ser universal neste grupo. O monitoramento da função renal, do crescimento e da vitalidade fetal deve ser frequente.

**Conclusão:**

A gravidez em pacientes transplantadas renais é desafiadora devido aos riscos maternos e perinatais. No entanto, esta revisão demonstra que o manejo clínico e obstétrico por uma equipe multidisciplinar experiente contribui para melhores desfechos maternos e perinatais nessas gestações.

## Introdução

A doença renal crônica (DRC) apresenta estimativas segundo as quais o estágio inicial afeta 3 em cada 100 gestações e o estágio avançado afeta 1 em cada 750 gestações, sendo que alguns desses pacientes evoluem com perda da função renal e necessitam de terapia de substituição renal, como hemodiálise e transplante renal^1,2^.

Pacientes com DRC podem apresentar bloqueio do eixo hipotálamo-hipófise-ovariano, com redução dos níveis sanguíneos de estrogênio e da depuração de prolactina, levando à anovulação, amenorreia e infertilidade. No entanto, com os avanços nas técnicas de diálise e o aumento da taxa de transplantes renais, as taxas de concepção tornaram-se mais frequentes^3^. As taxas de fertilidade em mulheres submetidas a transplantes renais e à hemodiálise são de 1/10 e 1/100, respectivamente^4^.

O cuidado com gestantes submetidas a transplante renal representa um desafio, não apenas pela compreensão das alterações fisiológicas do sistema renal durante a gravidez, mas também pelo risco de rejeição do enxerto, além do maior risco de comorbidades concomitantes, como distúrbios cardiovasculares e diabetes^5,6^.

Meta-análises reiteram o impacto da doença renal durante a gravidez, com uma série de complicações; portanto, é importante compreender o melhor momento para a concepção, bem como o manejo clínico e obstétrico mais apropriado, para otimizar o prognóstico materno e fetal após o transplante renal^5,7^.

Embora seja considerada uma situação de alto risco obstétrico, a gravidez em pacientes transplantadas renais não é contraindicada. No entanto, requer orientação adequada para que a concepção ocorra em um momento de estabilidade clínica da doença subjacente e de outras comorbidades, com o uso de imunossupressores em doses seguras, visando reduzir o risco de comprometimento da função renal e da consequente perda do enxerto. Além disso, o acompanhamento multidisciplinar com obstetras, nefrologistas, nutricionistas e neonatologistas é essencial para a vigilância e o manejo adequado de gestantes com transplante renal^8^.

Apesar do aumento da taxa de fertilidade em receptoras de transplante renal, ainda existem evidências limitadas, especialmente em relação ao manejo clínico e obstétrico adequado para a obtenção de melhores resultados obstétricos e neonatais^8,9,10^. Portanto, o objetivo desta revisão é explicar as recomendações atuais relacionadas à gravidez em mulheres com transplante renal, destacando novos consensos e esclarecendo dúvidas sobre o manejo clínico e obstétrico, bem como detalhes do tratamento, com vistas à redução de possíveis complicações.

## Método

Para elaborar esta revisão integrativa, foram incluídos estudos que abordassem gravidez e transplante renal, com foco no manejo clínico e obstétrico. Foram incluídos estudos disponíveis no PubMed e na base de dados SciELO, publicados até dezembro de 2024. Os seguintes descritores foram utilizados: gravidez; transplante renal; doença renal crônica. Os resumos de 354 artigos foram avaliados, dos quais 76 foram selecionados, incluindo metanálises e revisões sistemáticas. Foram priorizados os artigos que abordassem os seguintes aspectos: aconselhamento pré-concepcional na gravidez pós-transplante, manejo clínico e obstétrico e complicações maternas e perinatais, piora da função do enxerto durante a gravidez e cuidados pós-parto e aleitamento materno.

### Fertilidade e Concepção Após Transplante Renal

Além da recuperação da função renal, um dos principais benefícios do transplante renal para mulheres com DRC é a recuperação da função reprodutiva, sexual e da fertilidade, que ocorre aproximadamente seis meses após o transplante^11,12^. A gravidez em receptoras de transplante renal é relativamente rara, aproximadamente 5 casos por 100.000 nascimentos^11^. O transplante pode aumentar em até 10 vezes a chance de nascimento vivo em mulheres grávidas que recebem transplante, em comparação com aquelas em diálise^12,13^.

A Sociedade Americana de Transplantes, a Sociedade Italiana de Nefrologia, a Diretriz Europeia de Boas Práticas e a KDIGO (Doença Renal: Melhorando os Resultados) sugerem que as mulheres devem ser aconselhadas a esperar pelo menos um ano após o transplante^9,10,14^ e, de preferência, até dois anos, para engravidar, a fim de evitar disfunção do enxerto, rejeição ou falência, infecções e efeitos teratogênicos dos imunossupressores^10,14,15^.

A Sociedade Americana de Transplante Renal^14,15^ estabelece as seguintes recomendações para uma gravidez com menor risco de complicações:

- Ausência de episódios de rejeição por pelo menos um ano;
- Função renal estável (creatinina sérica < 1,5 mg/dL) e proteinúria ausente ou mínima (< 500 mg/24 h);
- Ausência de infecções que possam ser prejudiciais ao feto (como citomegalovírus e toxoplasmose);
- Utilização de imunossupressores que não sejam teratogênicos;
- Consideração de fatores adicionais: idade materna; adesão terapêutica; comorbidades que podem afetar a gravidez e a função do transplante^14,15^.

Especialistas recomendam que mulheres com DRC esperem a realização de um transplante renal antes de tentar engravidar. No entanto, deve-se enfatizar que, mesmo com o transplante, a gravidez permanece com alto risco de complicações (pré-eclâmpsia, infecções oportunistas, prematuridade, restrição de crescimento fetal [RCF] e mortalidade perinatal), exigindo acompanhamento rigoroso durante o pré-natal e supervisão por uma equipe multidisciplinar experiente^16^.

### Segurança Dos Imunossupressores e Outros Medicamentos

Os imunossupressores são usados em mulheres grávidas com doenças autoimunes (lúpus, glomerulonefrite primária e outras), para controlar a doença e manter a remissão, bem como em receptores de transplante renal, para evitar a rejeição do enxerto^17^. Os imunossupressores atravessam a barreira placentária e, portanto, é essencial avaliar o risco de teratogênese e de toxicidade fetal^10^.

O micofenolato de mofetila e a ciclofosfamida são teratogênicos e devem ser substituídos, idealmente, pelo menos três meses antes da gravidez^18,19^. Esses fármacos podem aumentar o risco de malformações, como anomalias faciais, cardíacas e viscerais congênitas e hérnia diafragmática, além de aumentar o risco de aborto espontâneo^17,20,21,22^.

Os corticosteroides, a azatioprina e os inibidores da calcineurina (ciclosporina e tacrolimo) apresentam um bom perfil de segurança na gravidez^17^. A azatioprina pode causar supressão grave da medula óssea em algumas gestantes receptoras de transplante renal, sendo necessário monitoramento rigoroso^20,23^. A farmacocinética dos inibidores da calcineurina se altera durante a gravidez, exigindo um aumento de 20–25% na dose para atingir a concentração adequada^24^. O sirolimo e o everolimo são contraindicados durante a gravidez devido ao seu efeito antiproliferativo, que pode afetar o desenvolvimento fetal^24^.

Dentre os corticosteroides, a prednisona é considerada a mais segura, pois apenas uma pequena fração atravessa a barreira placentária. Por outro lado, mulheres que utilizam doses superiores a 1–2 mg/kg/dia durante a gravidez ou próximo ao parto podem apresentar supressão da função do eixo hipotálamo-hipófise-adrenal, sendo recomendada a administração de corticosteroides intravenosos no momento do parto para evitar complicações. O uso crônico de corticosteroides aumenta o risco de insuficiência adrenal, hipertensão e diabetes gestacional. Além disso, há risco de hipoplasia do timo na infância. Por todos esses motivos, seu uso deve ser feito com cautela^17,25^.

Agentes imunobiológicos—como rituximabe, eculizumabe e belimumabe—diferem em estrutura e meia-vida; embora a passagem pela placenta seja limitada durante a organogênese, essa passagem aumenta exponencialmente ao longo da gravidez^26^. As informações sobre a segurança desses agentes na gravidez são escassas, e existem poucas recomendações e estudos específicos no período pré-concepcional, na gravidez e na lactação.

A hidroxicloroquina é um imunomodulador usado no lúpus para prevenir crises, com dados de segurança bem estabelecidos durante a gravidez e a lactação; atravessa a barreira placentária, mas não está associada à toxicidade fetal. Há também evidências de melhora da placentação e de prevenção de RCIU (Restrição de Crescimento Intrauterino), bem como de bloqueio atrioventricular e de lúpus congênito^19,27^.

As escolhas de medicamentos devem ser individualizadas, considerando os riscos de infecção oportunista, malformações estruturais, aborto espontâneo e parto prematuro, em contraposição ao risco de recorrência da doença materna devido à interrupção do uso desses agentes terapêuticos^20,26^. A Tabela 1 apresenta os imunossupressores e medicamentos utilizados em pacientes com DRC e transplante renal e sua segurança durante a gravidez e lactação.

**Tabela 1:** Imunossupressores e outros medicamentos utilizados em pacientes com drc e transplante renal durante o ciclo gravídico-puerperal

DROGAS	Concepção Seguro/Inseguro	Seguro/Inseguro	Gravidez e Efeitos maternos	Efeitos fetais	Lactação Seguro/Inseguro
DROGAS IMUNOSSUPRESSORAS					
Corticosteroides	Seguro	Seguro (C)	Maior risco de perda óssea, diabetes, infecção e ruptura prematura de membranas.	Sem teratogenicidade (estudos recentes não demonstraram associação com fenda orofacial).	Seguro (monitorar recém-nascido)
Azatioprina	Seguro	Seguro (D)	Supressão da medula óssea e hepatotoxicidade.	Não há metabolização pelo fígado fetal.	Seguro
Ciclosporina	Seguro	Seguro (C)	Risco de efeitos hipertensivos, hiperglicêmicos e nefrotóxicos.	Sem teratogenicidade (relatos de RCIU e recém-nascido pequeno para a idade gestacional).	Seguro
Tacrolimo	Seguro	Seguro (C)	Semelhante à ciclosporina.	Sem teratogenicidade.	Seguro
Hidroxicloroquina	Seguro	Seguro (C)	A abstinência pode causar crise de lúpus.	Sem teratogenicidade.	Seguro
A SER EVITADO					
Inibidores de mTOR (Sirolimo e Everolimo)	Inseguro	Inseguro (C)	Hiperlipidemia, hiperglicemia e nefrotoxicidade.	Não está claro (tóxico em estudos com animais).	Não está claro
Micofenolato	Inseguro (♀ e ♂: interromper > 6 semanas antes da concepção).	Inseguro (D)	Sintomas gastrointestinais e relação dose-resposta. Supressão de medula óssea.	Teratogênico. Malformações cardiovasculares e cranianas fetais graves.	Inseguro (excretado no leite materno)
Metotrexato	Inseguro (interromper > 3 meses antes da concepção).	Inseguro (X)	Hepatotoxicidade, sintomas gastrointestinais, alopecia e supressão da medula óssea.	Teratogênico.	Inseguro (excretado no leite materno)
Ciclofosfamida	Inseguro (♀ e ♂: interromper o uso >3 meses antes da concepção).	Inseguro (D)	Afeta a função ovariana e a fertilidade.	Teratogênico. Anomalias congênitas do crânio, orelhas, face, membros e órgãos viscerais; citopenia.	Inseguro (excretado no leite materno; suspender a amamentação durante o tratamento e por 36 horas após o término).
AGENTES BIOLÓGICOS				
Rituximabe	Não está claro (o fabricante recomenda interromper o uso 1 ano antes da concepção).	Não está claro	Passagem ativa no 2º e 3º trimestres. Administrar antes ou no início da gravidez.	Evitar, a menos que os benefícios potenciais superem os riscos (riscos potenciais de depleção de células B neonatais).	Não está claro
Eculizumabe	Não está claro	Não está claro	Monitorar a necessidade de aumento da dosagem.	Passagem ativa no 2º e 3º trimestres. Não foram relatados casos de teratogenicidade. Estudos realizados até o momento não demonstraram teratogenicidade.	Não está claro
Belimumabe	Não está claro (interromper >4 meses antes da concepção).	Não está claro	Passagem ativa no 2º e 3º trimestres. Administrar antes ou no início da gravidez.	Dados limitados. Evitar, a menos que os benefícios potenciais superem os riscos.	Não está claro
OUTRAS DROGAS					
Aspirina	Seguro	Seguro (C)	Diminui o risco de pré-eclâmpsia e restrição de crescimento fetal.	Sem teratogenicidade.	Seguro
Heparina de baixo peso molecular	Seguro	Seguro (C)	Diminui o risco de tromboembolismo venoso (TEV).	Sem teratogenicidade.	Seguro
Alopurinol	Seguro	Seguro (C)	Nenhum	Sem teratogenicidade.	Excretado no leite materno. Não há relatos de efeitos adversos.
Ferro	Seguro	Seguro (B)	Nenhum	Sem teratogenicidade.	Seguro
Eritropoietina	Seguro	Seguro (C)	Risco de hipertensão	Sem teratogenicidade.	Seguro
AGENTES BIOLÓGICOS					

Nota – Modificado de Gouveia et al.^51^.

Armenti et al.^28^ relataram uma taxa de 3–5% de malformações congênitas em receptores de transplante renal. No entanto, não foi observada associação com malformações congênitas relacionada ao uso de ciclosporina e tacrolimo^11,28^.

### Acompanhamento Obstétrico e Cuidados Pré-natais em Pacientes Transplantadas Renais

No acompanhamento pré-natal, as consultas devem ser mais frequentes, a cada 3–4 semanas até as 28 semanas; quinzenalmente entre as 28 e 34 semanas e, posteriormente, semanalmente. As avaliações do crescimento e desenvolvimento fetal devem ser rigorosas, com ultrassonografias seriadas. A partir das 20 semanas, deve-se realizar a avaliação com velocimetria Doppler e, após as 34 semanas, incluir o perfil biofísico fetal e a cardiotocografia, para monitorizar o bem-estar fetal^29^.

Deve-se realizar rastreamento rigoroso de anemia, com dosagem mensal de hemoglobina e hematócrito, instituindo-se o tratamento com sais de ferro e eritropoietina quando necessário. Além disso, recomenda-se a suplementação com ácido fólico na dose de 5 mg/dia para prevenir defeitos do tubo neural no primeiro trimestre. Posteriormente, a suplementação com ácido fólico deve ser mantida durante toda a gravidez, combinada com cianocobalamina, na dose de 1–2 mg/dia, para prevenir anemia megaloblástica^8^.

Recomenda-se o uso de ácido acetilsalicílico (AAS), em baixas doses, de 81–150 mg, em gestantes com transplante renal. Deve-se iniciar a partir da 12ª semana e suspender na 36ª semana de gestação, para reduzir o risco de pré-eclâmpsia precoce e melhorar a placentação em pacientes com fatores positivos para autoimunidade^19,30,31^. Em países com baixa ingestão de alimentos ricos em cálcio, a suplementação de cálcio de 1–1,5 g/dia é indicada para reduzir o risco de pré-eclâmpsia^19^.

Em mulheres grávidas com lúpus, portadoras de anticorpos antifosfolipídicos reativos, além do AAS, é necessário associar heparina de baixo peso molecular, em dose profilática, a fim de prevenir resultados obstétricos e fetais adversos^32,33^.

Estima-se que haja um aumento de 14,6% a 42% no risco de infecção do trato urinário nessas mulheres, bem como um risco aumentado de progressão para formas graves de pielonefrite. Portanto, deve-se realizar urocultura mensal. Dessa forma, recomenda-se o uso de antibióticos profiláticos após o primeiro episódio de infecção do trato urinário para prevenir a recorrência^7^. A avaliação da função renal deve ser precoce e seriada (mensal), com dosagem dos níveis séricos de ureia, creatinina e proteinúria de 24 horas ou da relação proteína/creatinina^34^. Além disso, a realização mensal de PCR para DNA do CMV é recomendada, visto que estudos demonstram que cerca de 40% das infecções por CMV em gestantes imunossuprimidas resultam em transmissão fetal, com 10% a 15% apresentando envolvimento grave^35.^ Caso ocorra soroconversão ou reativação da infecção por CMV durante a gestação, recomenda-se o tratamento com ganciclovir ou valaciclovir^36^.

As vacinas vivas atenuadas são contraindicadas em pacientes transplantados de órgãos sólidos. No entanto, a vacinação de mulheres grávidas com transplante renal é semelhante à das demais gestantes, sendo recomendadas vacinas contra influenza, hepatite B, difteria, tétano e coqueluche acelular (DTPa)^37^.

O parto vaginal não é contraindicado devido à localização do rim na cavidade pélvica. No entanto, estudos revelam altas taxas de cesariana em pacientes transplantadas renais, principalmente devido a complicações como pré-eclâmpsia e RCF^5,13^. A via de parto é indicada por critérios obstétricos, e sugere-se que a indução seja realizada a partir das 38 semanas, caso não haja complicações obstétricas^5,38^.

Quando uma cesariana é indicada, deve ser realizado um ultrassom abdominal para avaliar a localização correta do enxerto, com planejamento cirúrgico adequado, evitando trauma ao enxerto e ligadura inadvertida do ureter, que pode ocorrer em até 2% dos casos^39^.

Assim, apesar dos riscos envolvidos, a maioria dos estudos indica que é possível ter gestações bem-sucedidas após transplante renal. Os principais comportamentos que resultam em melhores desfechos maternos e perinatais incluem:

- Monitoramento rigoroso da função do rim transplantado;
- Rastreamento e tratamento adequado de infecções, principalmente do trato geniturinário;
- Início precoce de medicamentos que podem prevenir a pré-eclâmpsia (AAS e sais de cálcio);
- Controle rigoroso dos níveis de pressão arterial;
- Uso de imunossupressores seguros em doses ajustadas;
- Supervisão do crescimento fetal e vigilância do bem-estar^8^.

Na Figura 1, apresentamos uma estrutura esquemática do manejo clínico e obstétrico em gestantes com transplante renal.

**Figura 1:** Manejo clínico e obstétrico em gestantes com transplante renal.

Fonte: Preparado pelos autores

### Complicações Maternas Notransplante Renal

Um dos principais preditores do prognóstico e dos desfechos materno-fetais é a ausência de função renal pré-gestacional estável, com creatinina sérica elevada (> 1,5 mg/dL)^13,40,41^.

Em receptoras de transplante renal, a taxa de nascidos vivos é próxima de 75–80%, maior do que na diálise. No entanto, apesar da restauração da função renal, essas gestações ainda apresentam maior incidência de complicações^12,42,43^.

As complicações mais comuns são: hipertensão (24,2–54,2%); pré-eclâmpsia (21,5–31%); prematuridade (43,1–65,4%); recém-nascido pequeno para a idade gestacional (54%); rejeição aguda do enxerto durante a gravidez (1–9,4%) e no pós-parto (1,3%)^10,12,42,43,44^.

Além disso, taxas mais elevadas de cesarianas são relatadas (53–72%)^43,44^. Essas taxas variam muito em coortes internacionais sobre transplantes e gravidez, como a coorte americana de Shah et al.^44^ (35,4%) e a coorte chinesa de Ma et al.^45^ (94,1%).

Em gestantes com transplante renal, o diagnóstico de pré-eclâmpsia é complexo e desafiador, visto que a maioria dessas pacientes já apresenta hipertensão e é comum o desenvolvimento de proteinúria durante a gestação devido à doença de base, mesmo na ausência de pré-eclâmpsia^46,47,48^. Stepan et al.^48^ demonstraram que a angiotensina II promove a produção da tirosina quinase 1 solúvel semelhante a fms (sFlt-1) em células tubulares proximais. Evidências sugerem que o uso desses biomarcadores, associados ao Doppler do fluxo uteroplacentário, pode ser uma ferramenta auxiliar para o diagnóstico diferencial de pré-eclâmpsia em gestantes com DRC. Contudo, devido aos custos e à limitada disponibilidade para a prática clínica, são necessários mais estudos^48^.

Chewcharat et al.^16^, analisando dados de sua coorte de 295 gestantes com transplante renal e 405 gestantes com DRC em estágios 3–5, identificaram uma prevalência ligeiramente menor de pré-eclâmpsia (34,6%) em gestantes com transplante renal, em comparação com aquelas em estágios finais de DRC (48,4%), mas maior do que a de gestantes sem doença renal (4,4%). Os autores também concluíram haver três vezes mais chances de prematuridade e duas vezes mais chances de RCIU em receptoras de transplante renal^16^. Da mesma forma, uma metanálise de Shah et al.^44^ demonstrou uma taxa de pré-eclâmpsia seis vezes maior em receptoras de transplante renal do que na população geral dos Estados Unidos da América (21,5% versus 3,8%)^16,44^. Em relação aos dados sobre gravidez em receptoras brasileiras de transplante renal, o estudo de Radaelli et al.^49^ demonstrou uma taxa mais alta de pré-eclâmpsia (42%).

Para mulheres que foram submetidas a transplante renal na infância, pode haver associação com malformações renais congênitas e, portanto, alterações no trato urogenital devem ser excluídas. Nesse sentido, alterações nas cavidades uterina e cervical devem ser monitoradas, pois podem aumentar o risco de parto prematuro espontâneo^50^, visto que as receptoras de transplante renal já apresentam um risco 30,6% maior de parto com menos de 37 semanas do que a população em geral^11^.

Devido ao uso de imunossupressores e corticosteroides, as pacientes transplantadas renais apresentam maior risco de infecções do trato urinário (ITU), corioamnionite e endometrite do que outras gestantes^11,51^. Estudos demonstraram um risco mais elevado (14,6% a 42%) de ITU^51,52^. Uma metanálise recente de Souza et al.^52^ revelou que a taxa de prevalência de ITU entre gestantes latinoamericanas foi de 7,54%, sendo ligeiramente maior quando considerada a bacteriúria assintomática, estimada em 18,45%^52^. A infecção do trato urinário e a pielonefrite aguda são as principais complicações infecciosas nessas mulheres. Portanto, o tratamento deve ser assertivo, pois reduz o baixo peso ao nascer e o risco de prematuridade. É imprescindível avaliar a função renal durante a antibioticoterapia e após uma semana de tratamento^53^.

Akcay et al.^42^ compararam mulheres transplantadas que engravidaram mais de dois anos após o transplante com mulheres transplantadas que não engravidaram. Das 29 gestações, 72,4% resultaram em recém-nascidos vivos, e 38% das pacientes foram diagnosticadas com pré-eclâmpsia. Por outro lado, 33% apresentaram proteinúria > 300 mg/dL, mas não desenvolveram pré-eclâmpsia. Quase todos os partos (90%) foram cesarianas, com 38% de partos prematuros e 19% dos recém-nascidos pequenos para a idade gestacional. Observou-se uma probabilidade cinco vezes maior (IC 95%: 1.247–20.781; p < 0,02) de piora da função renal após um ano da gestação, em comparação ao grupo controle, com uma queda de pelo menos 25% na taxa de filtração glomerular, sugerindo que a gravidez compromete a função renal do enxerto a longo prazo^42^.

Metanálises sugerem que a gravidez que ocorre no primeiro ano após o transplante está associada a piores resultados maternos^54,55^. Por outro lado, estudos australianos e neozelandeses não demonstraram aumento na mortalidade durante o ciclo gravídico-puerperal^46^. A Figura 2 apresenta as principais complicações maternas observadas em gestantes com transplante renal.

**Figura 2:** Complicações maternas e perinatais em pacientes

Fonte: Preparado pelos autores.

### Complicações Perinatais em Receptores de Transplante Renal

Em comparação com a população em geral, as gestações em mulheres com transplante renal apresentam taxas mais elevadas de recém-nascidos prematuros, pequenos para a idade gestacional e com baixo peso ao nascer, levando a internações mais frequentes na UTI neonatal. Assim, tais desfechos resultam em taxas mais elevadas de morbidade e mortalidade perinatal nessas mulheres^7,41,56,57,58^. Além disso, a exposição intrauterina a imunossupressores contribui para o aumento de infecções no primeiro ano de vida^5^.

O Registro Nacional de Transplantes dos Estados Unidos demonstrou taxas de nascidos vivos em receptores de transplante renal variando de 71–76%^56^. Coortes internacionais, como a de Shah et al.^44^ (72,9%), Mustafa et al.^59^ (78,5%) e a coorte brasileira de Radaelli et al.^49^ (56,7%), apresentaram resultados semelhantes.

Em estudos realizados nos EUA com receptoras de transplante renal, os nascimentos com menos de 37 semanas variam de 43,1% a 65,4%^7,44^. Shah et al.^44^ demonstraram que as taxas de prematuridade neste grupo são mais elevadas na América do Sul (55%) e mais baixas na América do Norte (35,4%)^7,44^.

O peso ao nascer de filhos de mães transplantadas renais é, em média, menor do que o da população geral dos EUA: 2470 g versus 3389 g^7,44^. Por outro lado, Arab et al.^11^ demonstraram uma alta taxa de RCIU (30%). A anemia materna é comum em pacientes transplantadas renais e pode ser uma das explicações para essa associação com a RCIU. Além disso, mesmo com o transplante renal, a progressão da vasculopatia em pacientes com diabetes e vasculite sistêmica não é reduzida, o que pode contribuir para a RCIU e para o baixo peso ao nascer^11^. Estudos demonstram que as taxas de recém-nascidos com baixo peso ao nascer (peso < 2500 g) estão entre 43% e 54% nesses pacientes^42^. Shah et al.^44^ também concluíram que existe uma probabilidade três vezes maior de prematuridade e duas vezes maior de restrição do crescimento fetal em receptoras de transplante renal^44^.

O estágio da DRC e a hipertensão são determinantes importantes dos desfechos^41^. Pacientes transplantados renais com taxa de filtração glomerular estimada superior a 90 mL/min/1,73 m^2^ apresentam piores desfechos em comparação com pacientes com DRC estágio 1; essas diferenças se estabilizam quando apenas pacientes com DRC afetados por glomerulonefrite ou doenças sistêmicas (“DRC progressiva”) são comparados com pacientes transplantados renais. Na análise multivariada, o risco de parto prematuro e de parto prematuro extremo foi associado ao estágio da DRC (2–5 vs. 1: risco relativo [RR] 3,42 e 3,78, respectivamente) e à hipertensão (RR 3,68 e 3,16, respectivamente), enquanto nenhuma diferença foi associada ao fato de o paciente ser transplantado renal ou ter DRC^41^.

Estima-se que o risco de aborto espontâneo seja menor do que o da população em geral (14–15,4% versus 17,1%)^11^. As mortes perinatais variam de 2,5% a 5,1%, uma taxa superior à da população em geral nos EUA (0,6%)^7,44^. A taxa de mortalidade perinatal é quatro vezes maior em receptoras de transplante renal do que na população em geral, o que pode ser atribuído à RCF e à prematuridade^18^. Essa taxa é maior em pacientes que engravidam nos primeiros dois anos após o transplante, em comparação com aquelas que engravidam posteriormente^60^. A Figura 2 apresenta as principais complicações perinatais observadas em gestantes com transplante renal.

Bachmann et al.^61^ acompanharam filhos de mulheres com transplante renal desde o nascimento até os dois anos de idade e demonstraram que as medidas antropométricas apresentaram evolução semelhante, tanto em nascimentos prematuros quanto a termo, e sem alterações no desenvolvimento neuropsicomotor^61^. Nulman et al.^62^ também não encontraram atrasos no desenvolvimento neuropsicomotor ou no comportamento em crianças acompanhadas até os oito anos de idade expostas à ciclosporina intrauterina, em comparação com controles femininos saudáveis^62^.

### Piora da Função do Enxerto Durante a Gravidez

A gravidez em mulheres com função renal estável não aumenta o risco de rejeição do enxerto^63^. Por outro lado, receptoras de transplante renal sem função renal estável apresentam risco de rejeição aguda de 1,3% a 9,4%^47,63^ e de perda do enxerto de 5,6% a 9,2%^44,63^ após a gravidez, especialmente se esta ocorrer em menos de um ano após o transplante^10^. A literatura recente demonstra que taxas mais elevadas de perda da função do enxerto ocorrem em mulheres com creatinina sérica > 1,5 mg/dL. Portanto, o aconselhamento de receptoras de transplante renal sobre a longevidade do enxerto é essencial ao se considerar a gravidez^13,40^.

O uso de imunossupressores em dose adequada está diretamente relacionado à manutenção da função estável do enxerto, com menor probabilidade de rejeição. A ciclosporina e o tacrolimo são dois dos imunossupressores mais utilizados e devem ser administrados em doses seriadas durante a gravidez, pois seus níveis séricos podem diminuir devido à hemodiluição e ao aumento do metabolismo hepático, exigindo reajustes para atingir concentrações terapêuticas. A dosagem sérica deve ser realizada mensalmente no primeiro e no segundo trimestres e semanalmente no terceiro trimestre da gestação^64^.

Akcay et al.^42^ demonstraram que a piora da função do enxerto renal a longo prazo está relacionada à persistência de níveis elevados de proteinúria nos primeiros três meses do pós-parto, bem como a níveis elevados de creatinina sérica nos primeiros seis meses do pós-parto. Além disso, o tempo decorrido entre o transplante e a concepção parece influenciar o risco de deterioração da função renal^42^.

Registros do Centro Nacional de Transplantes dos Estados Unidos indicam que mulheres com perda da função renal durante ou após a gravidez apresentam valores de creatinina pré-concepcional mais altos do que aquelas com função renal estável (1,5 ± 0,6 versus 1,3 ± 0,4 mg/dL). Por outro lado, mulheres afro-americanas apresentam maior risco de perda da função renal se engravidarem em menos de dois anos após o transplante (13%), em comparação com mulheres brancas (5%)^65^.

A hipertensão arterial pré-concepcional parece ser um dos principais preditores de declínio da função renal e do risco de rejeição a longo prazo. Estima-se que 25–40% das mulheres com transplante de órgãos sólidos já sejam hipertensas antes da gravidez^39^. Alguns fatores relacionados a esse fato seriam a toxicidade vascular das calcineurinas, a hipertensão induzida por corticosteroides e o efeito vasopressor resultante da isquemia renal^64,66^.

Os níveis de creatinina são significativamente mais elevados em mulheres hipertensas antes da gestação^67,68^. A disfunção do enxerto moderada a grave é definida por creatinina > 1,4 mg/dL e > 1,9 mg/dL, respectivamente^14,65,69^. A proteinúria > 1 g/24 h, antes ou durante a gravidez, também está relacionada a um maior comprometimento da função do enxerto^70^.

Durante a gravidez, a principal causa de elevação da creatinina sérica é a presença de pré-eclâmpsia. No entanto, também pode estar relacionada à infecção e à obstrução do trato urinário ou à toxicidade por tacrolimo. Seja como for, qualquer aumento na creatinina deve ser imediatamente investigado e tratado adequadamente para evitar a piora progressiva da função renal e o risco de perda do enxerto^15^.

Mulheres com transplante renal e painel elevado de anticorpos anti-HLA (antígeno leucocitário humano) específicos do doador constituem um grupo com maior risco de disfunção renal e perda do enxerto. Não se sabe se a gravidez é um fator desencadeante desse processo ou se o mesmo está relacionado ao tempo decorrido desde o transplante^60^.

A avaliação dos rins pode ser aprimorada pela análise do fluxo sanguíneo renal com Dopplervelocimetria dos vasos do enxerto, medindo a velocidade do fluxo e o índice de resistência (IR). Em rins com função preservada, essa resistência diminui ao longo da gravidez, principalmente entre as 13 e 16 semanas, com tendência a aumentar no final da gestação. Valores de IR > 0,8 apresentam prognóstico desfavorável e podem indicar o desenvolvimento de nefropatia no enxerto, obstrução renal, pielonefrite ou pré-eclâmpsia, exigindo investigação adicional e uma abordagem assertiva para evitar danos ao enxerto^8^.

A taxa de rejeição do transplante durante a gravidez é baixa, em torno de 4,2% na população americana, segundo uma metanálise recente^5^. Durante a gravidez, existe um mecanismo de tolerância imunológica fisiológica, com o envolvimento de moléculas HLA-G, que inibem linfócitos T, células *natural killer* e células apresentadoras de antígenos^24^. Se houver suspeita de rejeição, a biópsia renal deve ser considerada após a viabilidade fetal (> 25 semanas) e, se a idade gestacional for avançada (> 34 semanas), sugere-se tratamento empírico com metilprednisolona e consideração da interrupção da gravidez^46^.

### Cuidados Pós-Parto e Amamentação

Estudos evidenciam um risco dobrado de hemorragia pós-parto (HPP) e de necessidade de transfusões sanguíneas em mulheres no pós-parto com transplante renal. Existem teorias que sugerem que os imunossupressores têm um efeito prejudicial na remodelação das arteríolas uterinas, reduzindo a contratilidade e a miotamponação, aumentando assim o risco de HPP^71,72^. Além disso, sugere-se o uso de antibióticos após a cesariana, devido ao maior risco de infecção associado ao uso de imunossupressores^11^.

Há evidências de excreção de medicamentos imunossupressores no leite materno; no entanto, os benefícios da amamentação parecem superar os riscos da exposição^73^. Tacrolimo, ciclosporina, azatioprina e prednisona são considerados seguros durante a gravidez e a amamentação^71,72,73^.

### Aconselhamento Pré-Concepcional

A contracepção deve ser recomendada para todas as mulheres com DRC em idade reprodutiva que sejam sexualmente ativas e que não desejem engravidar. Os contraceptivos combinados com estrogênio são contraindicados devido ao aumento do risco cardiovascular e tromboembólico. O acetato de medroxiprogesterona deve ser evitado devido à possível redução da massa óssea. Pílulas contendo apenas progestagênio, implantes subdérmicos de etonogestrel e dispositivos intrauterinos são recomendados^1,74^.

Não há evidências que sustentem que os dispositivos intrauterinos sejam menos eficazes em receptores de transplante renal devido à imunossupressão, nem que estejam associados a um maior risco de doença inflamatória pélvica^75^.

Uma consulta pré-concepcional com uma equipe multidisciplinar é essencial para ajustar imunossupressores, suplementação vitamínica e fornecer orientações adequadas sobre o prognóstico. Além disso, é necessário iniciar o pré-natal em um serviço terciário com uma equipe experiente no manejo de gestantes de alto risco.

A substituição de imunossupressores teratogênicos (micofenolato de mofetila e ciclofosfamida) por outros de classes mais seguras (azatioprina, ciclosporina e tacrolimo), e a suspensão de medicamentos embriotóxicos durante a gravidez, devem ser realizadas pelo menos 90 dias antes da concepção^76^.

Portanto, a orientação sobre os riscos de piora da função do enxerto, redução da sua longevidade e perda permanente da função do enxerto constitui um dos pilares essenciais do aconselhamento pré-concepcional^57^.

## Conclusão

A incidência de gravidez após transplante renal aumentou nas últimas décadas, e as tentativas de concepção devem ser planejadas pelo menos um ano após o transplante, na ausência de sinais de rejeição do enxerto, para permitir o ajuste da imunossupressão e reduzir o risco de perda do enxerto. O casal deve ser informado de que, embora as complicações maternas e perinatais sejam menores em receptoras de transplante renal do que em gestantes em hemodiálise, essas gestações apresentam maior risco de pré-eclâmpsia, prematuridade, baixo peso ao nascer, cesariana e óbitos perinatais. Contudo, mesmo sendo considerada uma gravidez de alto risco, se a concepção for planejada em um momento de estabilidade clínica materna, com imunossupressores seguros e acompanhamento obstétrico realizado por uma equipe multidisciplinar experiente, a chance de uma gravidez bem-sucedida torna-se um objetivo alcançável.

## Data Availability

Todas as informações desta revisão foram coletadas das referências utilizadas para a sua elaboração.
